# Human Rights in Chilean Social Work: Lessons from Chile to Prepare Social Work Students for Human Rights Practice

**DOI:** 10.1007/s41134-020-00156-8

**Published:** 2020-11-21

**Authors:** Gabriela Rubilar Donoso, Caterine Galaz Valderrama, Catherine A. LaBrenz

**Affiliations:** 1 Universidad de Chile Departamento de Trabajo Social, Santiago de Chile, Chile; 2 University of Texas at Arlington School of Social Work, Arlington, TX USA

**Keywords:** Human rights, Social work students, Memory, Testimonies, Chile

## Abstract

This article presents findings from two studies conducted in Chile to examine the link between human rights and social work practice. The focus of this paper was to explore the role of undergraduate education in preparing future social work practitioners for human rights practice. Data from a qualitative longitudinal study to understand the role of social workers during the dictatorship in Chile (1973–1989) were used; then, in October 2019, as civil unrest and police and military brutality erupted across the country, the authors created a commission to register and document narratives and testimonies of current human rights violations in Chile. The research team utilized a qualitative approach to analyze data from the in-depth interviews that were conducted in the longitudinal study and from the 2019 commission. Findings suggest a need to cover more in-depth human rights content in social work education and to teach students to create community collaborations in the field. Implications for social work education and practice in the current political climate are explored.

In October 2019, Chile experienced the greatest “social uprising since recovering democracy,” which had occurred thirty years prior (Garcés 2019; Araujo 2019). What began as a student protest against raising prices of public transit escalated into large-scale protest against neoliberalism and policies enacted during Pinochet’s dictatorship that have stratified and perpetuated inequities in Chilean society. In response, the government declared a *toque de queda* (curfew) and brought in the military to patrol the streets at night. As the protests and police and military presence escalated, several organizations registered instances of violations of human rights, police brutality, and abuse of power (Amnesty International 2020). The official Ministry of Health recognized that over 13,000 people were injured during the first two months of protests, and between October and December 2019, over 2500 formal reports were made alleging violations of human rights (Amnesty International 2020). Although the *toque de queda* declared in October was repealed after a few weeks, the global spread of coronavirus in March 2020 led to the government declaring *estado de excepción*, also giving police and military authorities more power to limit transit, mobility, and social organizing; this has led to concerns about disparities in enforcement and exposure, as well as concerns about extending policies that may prohibit forms of social protest. This paper presents the process that leaders from the School of Social Work at the *Universidad de Chile* undertook to document narratives of human rights violations during two main periods of unrest in Chile: The first of which was during Pinochet’s regime from 1973 to 1989 and the second from October 2019 until the present, during the *estallido social*. Findings from these processes have implications for social work practice and education and of social work practitioners as they enter the field.

Historically, social work has emerged across the globe as a response to social inequities and a commitment to human rights (Deegan 1997; Reininger 2018). In fact, women suffragists from the Hull House were also involved in the creation of formal social work and social justice (Álvarez-Uría and Parra 2014). In Latin America, this commitment to social justice has often remained even through periods of dictatorship (Morales 2015; Morales and Rodríguez, 2018; López 2018). While human rights are essential to global social work, they have particular relevance in the developing world and during Latin American dictatorships and periods of civil unrest, as these contexts have driven social work ethics and professional responsibility in addressing and remedying human rights violations (Moljo and Moljo 2006; Netto 1996; Quiroz 1985; Aguayo et al. 2018).

## Human Rights in Chilean Social Work

The first formal social work school in Chile emerged in 1925, shaped by its focus on human rights. One of the first schools of social work, *La Escuela Lucio Córdoba*, had courses starting in 1940 that emphasized the discipline’s commitment to dignity and human rights, especially for people experiencing social inequality. Prior scholars have noted “as social work was developed, ethics and human rights have been presented as essential and fundamental pillars of the discipline, even when these are not specifically named” (Matus et al. 2004, p. 356). During the period of the dictatorship that began in 1973, formal social work became divided into schools that shifted towards positivism, largely influenced be neoliberalist policies (Toledo 2004), and those that resisted the political shift and highlighted anti-oppressive social work practices (Healy 2001). Thus, although neoliberalism and policies under the dictatorship colonized knowledge production in some areas of social work, some schools and professionals became re-committed to resistance and human rights (Castañeda and Salamé 2013).

One consideration for human rights in social work practice in Chile is that many current practitioners are part of the “children of the dictatorship” generation. This “children of the dictatorship” generation represents people with one or more of the following characteristics: (1) those who were born during the democratic period that preceded the coup d’état in Chile, or who were born at any point during the 17 years of Pinochet’s dictatorship; (2) those who lived the majority or a large part of their childhood or adolescence during the dictatorship; (3) those who made a decision to study social work as the dictatorship ended between 1989 and 1990; or (4) those who began to study social work during the transition back to democracy. Many students during this period were motivated to study social work because of the discipline’s commitment to social justice and human rights.

Social workers who were part of the “children of the dictatorship” generation experienced three dynamic events that impacted their research trajectories: (1) a paradoxical process of transition to democracy (e.g., tension between principles of equality and that of individual responsibility), consequences of which are still present today (Araujo 2019; Cornejo et al. 2013); (2) the development of economic and social policies related to recuperation and expansion, which focused on reducing poverty levels and increasing development, yet maintained high levels of inequality; and (3) the change of millennium that brought with it important social transformation, including technological changes that changed ways in which information was produced, education was considered, and how knowledge was formed.

### Human Rights and Social Work During the Dictatorship

Prior literature has evidenced that the period between 1973 and the late 1980s consisted of a pioneer movement in social work in Chile as the discipline confronted a dialectical process of adapting to neoliberal policies while grappling with ongoing human rights violations (Healy 2001). While there was a push for more “objective,” micro-level interventions, several schools and professionals developed more formal ties to defending human rights and contributed to the return of democracy (Castañeda and Salamé 2010, 2013; Morales 2010, 2015; Morales 2018; Saracostti et al. 2014). This period not only shaped the identity of social workers throughout Chile but also reaffirmed and revalued the discipline and expanded its research and developmental scope. The discipline adapted its scope through creativity and seeking ways to serve victims in crisis in the aftermath of the Coup d’état (Del Villar 2018). Organizations responsible for halting serious human rights violations, especially during the first part of the dictatorship (1973–1979), had active collaboration with social workers from the beginning. Social workers led tasks related to aiding, registering, and documenting reports of disappearances and detentions that occurred throughout the country. Furthermore, social workers sought dignity for people who had been detained or disappeared and their families.

The main organization that oversaw human rights protections was the *Vicaría de la Solidaridad* and was comprised of teams of social workers, lawyers, and doctors, who were responsible for collecting information and reconstructing testimonies of families and victims of human rights violations. The *Vicaría de la Solidaridad* was created in Chile in 1976 to continue the work formerly conducted by the *Comité ProPaz*, a pro-peace committee that was dissolved by the dictatorship (Labbé and del Villar 2019). The *Vicaría de la Solidaridad* professionalized and systemized the documentation of human rights violations in Chile and later served as a reference for other countries in Latin America. Over the course of the dictatorship, the *Vicaría de la Solidaridad* created archives of more than 85,000 unique documents that have been essential to reconstruct the history of human rights violations in Chile and begin actions of reparation and the search for justice (Morales 2010; Bernasconi 2019). The process of documentation and registration implied, in many cases, adapting traditional social work practices and re-signifying them within the political context (Gallardo 1990; Del Villar 2018). Professional social workers utilized imagination, proximity, and interdisciplinary collaboration to create mechanisms to serve families, systems of registration, and innovative ways to produce knowledge and intervention, which led to a milestone in the debate of social work and human rights (Sánchez 1988, 1990, 2018). This made it possible to integrate traditional social work practices such as family interviews and home visits and to adapt them to a new context.

### Current Situation of Human Rights and Social Work in Chile

In the past decade, a new generation of students has emerged, and it is this new generation that was the protagonist of the October 2019 mobilization, the largest since the end of the Chilean dictatorship. Thirty years have passed since the “children of the dictatorship” were trained and began their professional social work careers. The new generation of social work students has been motivated by other concerns and has been able to develop their own trajectories without the weight and responsibility of political memory that impacted the previous generation. Yet, just as the previous generation experienced, this current generation has experienced serious violations of human rights and has actively sought out ways to support the defense and promotion of human rights and dignity.

In parallel, these new human rights violations and the social work response come within four years of the re-opening of social work at the *Universidad de Chile*, a school that was forced to close during Pinochet’s dictatorship and re-opened in 2015. Compared to past generations, current social work students must learn to practice in a context where the labor market has changed and become more flexible yet unpredictable. This includes large proportions of informal workers, working poor, and a high proportion of unstable jobs (Sehnbruch 2006), all of which may impact poverty and other issues among clients they will serve. Current social work students were born at the end of the past century, in a country with high levels of development and a member of the Organization for Economic Cooperation and Development (OECD) and assumptions about acknowledging and guaranteeing human rights. Yet, as the country continues to grapple with protests and inequality (Somma et al. 2020; Araujo 2019), social work students must prepare to address the political, social, and economic factors that have led to mass unrest and social outburst.

## Current Study

This article explores content related to Chilean social work and human rights over two distinct political periods separated by 30 years. The first period consists of the professional strategies and actions taken to address grave human rights violations during the Chilean dictatorship (1973–1989) and the researchers explore how this changed the role of the discipline. The second period consists of strategies and actions developed by academics and students during the ongoing political and social crisis that started with student protests in October, 2019. Through the use of in-depth interviews with individuals who experienced human rights violations, the narratives the team collected evidence human rights violations, and the researchers in charge of the project model ways for students to document, raise awareness, and address serious violations of human rights. In this context of post-dictatorship civil unrest, the current generation of social workers is being formed and as such, our discipline must prepare them to be champions of human rights.

### Theoretical Framework

The theoretical framework of generational agitation guides this study. This framework was developed by Mannheim (1993) and expanded upon by Castañeda and Salamé (2013) to explore the impact of historical, economic, and political factors on social movements over time. The social and political crisis in October, 2019, reflects a movement of generational agitation, in that it has impacted and mobilized different generations that have diverse experiences, knowledge, and professional skills and practices that need to be updated, adjusted, and adapted to new contexts. This intersection of generations at a joint collective action can lead to social movements. Social movements are driven by rejecting the status quo, disobedience, and actions as a form of resistance. Given the current social and political crises in Chile, the current social movement has connected current human rights violations and actions to address them to those in the dictatorship and during the transition to democracy (Artaza 2019; Matus 2019). Social movements emerge when individuals recognize that “individual suffering is not only shared suffering, but rather that an entire group has reached its limit” (Butler 2016, p. 24). Furthermore, collective social action includes both a rejection of established order and a desire to enact change (Didi-Huberman 2017). In this process, defense of human rights is re-established as a base to demand justice. Indeed, as the state continues to repress social movements during the *estallido social*, it reverts to the control and violence of the past, which, in turn, result in further resistance (Piper and Montenegro 2017). Thus, current social movements in Chile reflect past strategies to organize and defend human rights, while adding new strategies such as social media advocacy (Somma et al. 2020).

## Methods

Data from this article come from two recent studies that explored social work in Chile during periods of civil unrest and state-sanctioned violence. This included a longitudinal qualitative study (2014–2019) that explored research trajectories and transitions among Chilean social workers and an exploratory qualitative study in October–December 2019 to explore testimonies of human rights violations during the *estallido social* (political/social crisis)*.* For the first study, a qualitative longitudinal approach was used to follow professionals over time and compare perspectives within and between participants (Caïs et al. 2014). As such, the researchers analyze the written testimonies not only as a methodological product of the study but also as a practice of intergenerational memory. This includes viewing testimony as a democratic narrative form that assumes symbolic and cognitive value in narration of life experiences. In this approach, each individual testimony can give voice to the voiceless, their lives, and experiences (Beverley 2008). The research team utilized prior research on reflection on memory and dictatorship to guide this study design (Aguayo et al. 2018; Castañeda and Salamé 2010, 2013; González 2010, 2013, 2014; Morales 2010, 2015; Morales 2018; Rubilar 2018; Del Villar 2018). The goal of the study was to examine Chilean social work trajectories, with special emphasis on generational perspectives, including the authors’ own experiences and practices in human rights during the period immediately after the return to democracy (1991–1993), as well as subsequent actions related to studies on memory and defense of human rights during the civil unrest in October, 2019. The first study also included interviews from social workers that were formed as part of the “children of the dictatorship” and the most current generation that represents students currently studying social work or who have completed the degree in the past decade.

Starting in October, 2019, the authors began a parallel study that integrated diverse initiatives and interventions to promote and guarantee human rights in Chile. These include registration, reports, and visualization of human rights violations (INDH 2019). Among these, the authors created a Commission of Testimony and Narratives in October 2019. The commission consisted of students and academics at the *Universidad de Chile* and was sponsored by the chapter of the Professional College of Human Rights*.* With the collaboration of the Professional College of Human Rights, the researchers identified people and conducted interviews to victims or witnesses of violence or brutality perpetuated by the police force. Based on these intergenerational dialogues, the authors approached the following study as a way of preparing students to confront and address current human rights violations in the field and defend the discipline’s commitment to guaranteeing human rights and social justice. All of the testimonies from the longitudinal study and all of the testimonies compiled during the Commission’s work between October and December 2019 followed the Institutional Review Board protocol and rules that guide Chilean research with human subjects, and participants signed an informed consent protocol before beginning the interviews.

### Data Collection

The authors selected the narrative approach to collect data in both studies, due to previous research on those that disappeared or were detained during the dictatorship. The use of narratives has been shown to be therapeutic in some instances (White and Epston 1990) and can give voice (Bernasconi 2014, 2018; Sepúlveda 2005) to those who, in these cases, experienced state-sanctioned or state-perpetuated violence. In other instances, testimony and narrative can be used as a means of preserving memory, as was the case of Mónica Hermosilla and her last moments with María Teresa Bustillos, a social worker who was detained and disappeared at Villa Grimaldi in Chile (Illanes 2012). During the dictatorship, statements from witnesses and victims of repression served as a means of preserving memories (Fernández 1985; Sepúlveda 2005). Over time, legal investigations, journalism, and other documentation have allowed professionals to reconstruct history. This has led to a recognition of the role of professionals and interdisciplinary collaboration during the dictatorship. One example was the book *Archivo del cardenal*. *Casos reales* (Insunza and Ortega 2011, 2014) which turned into a series that followed a lawyer and social worker who collected testimonies through the *Vicaria de la Solidaridad*. During this period, it was usually social workers who were the first contact for families and compiled the information, while lawyers then sought legal action and protection (Del Villar 2018).

Utilizing a narrative approach, the authors of this study interviewed social workers as part of the longitudinal study to examine the link between human rights and social work over time. The first study included in this analysis began in 2014 and was longitudinal to collect data at three different time points (2014, 2015, and 2019) to understand social work research trajectories and transitions during key points in contemporary Chile. Data were collected via interviews, and participants were interviewed across time points. As the civil unrest erupted in 2019, the authors conducted a complementary study to explore experiences of human rights violations during the *estallido social*. In this complementary study, the authors also utilized a narrative approach to gather testimony both as a therapeutic means and as a way to preserve memory and document human rights violations of Chilean citizens and residents. Consistent with prior literature, testimonies were elaborated throughout the interview process with each participant (Rubilar 2015). All interviews conducted between October and December 2019 were recorded and transcribed verbatim. The students were trained and accompanied by the authors of this paper to compile and collect testimonies from professionals responsible for providing services to victims, witnesses of human rights violations, and/or victims of police repression that occurred between October and December, 2019.

### Sample

To date, the current paper includes 52 biographical interviews conducted among Chilean social workers from distinct generations in the longitudinal study, as well as 80 testimonies of police and military violence between October, 2019, and December, 2019, that were overseen by academics and 20 social work students at the *Universidad de Chile*. Thus, the sample consists of 52 Chilean social workers who have reflected on the role of human rights in social work during the dictatorship and/or transition to democracy and 80 narratives from Chilean adults who witnessed or experienced first-hand human rights violations during the *estallido social* that began in October, 2019, and has continued as we wrote this paper. To protect participant confidentiality, demographic information was not recorded.

### Data Analysis

This study utilized a qualitative approach to compile, analyze, and interpret narratives related to human rights violations in Chile during the dictatorship (1973–1989) and protests (October 2019–present). The authors integrate fragments from some of the compiled testimonies as a means of illustrating or displaying different themes from the analyses of the larger study. Applied thematic analysis was conducted to analyze the compiled testimonies. This approach was selected to explore the role of social work practitioners in documenting and addressing human rights violations in Chile. Prior literature has also utilized thematic analyses to explore social phenomena or issues relevant to social work (Chiarelli-Helminiak et al. 2018; Dapi et al. 2018). This involved two stages of identifying codes and emerging themes from the data (Guest et al. 2012). In the first stage, transcripts of the testimonies were read line-by-line and the team identified codes that emerged directly from the data. In the second stage, the team compared and contrasted codes to develop themes that captured broader ideas. All analyses were conducted with NVivo. Some strategies that the authors incorporated to increase trustworthiness of the data included training the team that collected the narratives, utilizing data from multiple sources, and supporting themes and interpretations with quotes (Guest et al. 2012).

### Positionality

In an effort to recognize the authors’ own experiences and identities and the impact of these on research (Bourke 2014), we provide the following reflection of our own positionality. Two of the authors of this paper have personal experience with human rights violations and social work, as part of the “children of the dictatorship” generation (Deckert et al. 2002; Cornejo et al. 2013). Although the third author is not directly part of the “children of the dictatorship” generation, she has family members that are and has worked extensively with individuals and families impacted by the dictatorship and its lasting consequences.

## Results

Based on the analysis of narratives form the longitudinal study of social workers and human rights, and the testimonies gathered between October and December 2019, two main themes emerged that were related to human rights and social work in Chile. The first theme was *incorporating human rights into social work curricula* and included suggestions for preparing students to advocate human rights as they enter the field. The second theme was *creating collaboration in the field* and focused on hands-on experiences and supports within the communities in which students begin their social work careers. Both themes are explored in detail below.

### Theme One: Incorporating Human Rights into Social Work Curricula

The first theme that emerged from the narratives was the need to continue to incorporate human rights information into social work curricula. Although participants reported some advances over the past thirty years, there was a need to integrate more in-depth material. For example, a testimony from a social worker reflecting on their work in the dictatorship reflects the challenges and perspectives that were intertwined in social work during this period, the lack of professional formation, and the importance of hands-on learning:

… All of this, I am learning as I go, I basically get support from my team. There were three lawyers, and we went into it learning together […] It was learning together, asking about the forms: “how do you do it? How do I store them?” Because there was a whole issue of security. How did you store it? How did you hide it? Honestly, we did not hide much, we did not store much. Well, we stored things in cardboard archives […] all of the registries were in folders, and there were a lot of risks and we were always afraid that they would get stolen or that someone would break into the office. We had double folders with all of the registries, but there was always this risk [of having them stolen]. I just got support from them […] I didn’t have any experience, I wasn’t even sure about how to handle the topic from the diocese, or about these issues. What we learned at the university was completely different and I think that all throughout Chile the same thing occurred. We did not have any elements, no methodology, so we had to learn in the field (testimony 31, compiled in 2014).

Thirty years later, this participant has developed a program to teach professionals about human rights. It is currently in its fourth version and includes materials for incorporating human rights materials into undergraduate courses in social work. In a follow-up interview, despite having incorporated content related to human rights in undergraduate courses, this participant reported a need to include more in-depth content on human rights:

… we are conducting research in schools of social work that are recognized by the Ministry of Education, and have been analyzing their content related to human rights, related to human rights and the gap that still exists between that content and the standards that the Institute for Human Rights has established […] So, our study examines what gaps exist. First, the course objectives, although they look nice on the syllabus, do not actually match the course content. The syllabus does not match what the instructor actually does. So, we made some modifications because at the beginning we had asked programs that we thought would have content related to human rights, whether they were explicit or implicit. And then what we did was we asked them to send us whatever syllabi they wanted” (testimony 31, compiled in 2019).

Over the course of thirty years, these two testimonies highlight advances in the formation of human rights in undergraduate social work programs. Now, many universities have developed material to address human rights, but there are still areas that need to be covered more in-depth and to compare with other perspectives. Table 1 below displays how the five highest-ranked universities in Chile have incorporated human rights material into their social work programs, based on the study reported in testimony 31.

**Table 1:** Human rights curricula in social work at the five universities that receive students with the highest college admission test scores

University	Type of course	Course name	Semester taken
Universidad de Chile	Required	Social justice, human rights, and citizenship	3rd semester
Pontificia Universidad Católica de Chile	Required	Human rights	7th semester
Universidad Alberto Hurtado	Content integrated into required courses	Poverty and social intervention	8th semester elective
		Ethics	
Universidad Católica de Valparaíso	Content integrated into required and elective courses	Social work ethics	8th semester elective
		Gender	
Universidad de Concepción	Elective	Social work with a human rights perspective	Elective

Source: Elaborated from information available on the internet

Participants reported that the majority of universities had clearly listed integration of human rights among competencies for social work students, but there was no consensus about how to measure this or which specific strategies social work educators could take to ensure students met this competency. In many cases, human rights material is integrated into other courses, such as direct practice, ethics, or other specialization courses. Across universities, preparation in human rights requires effort from the instructor, students, and people in the local communities with whom they will interact.

One particular example that emerged from our study was the undergraduate curricula developed by the *Universidad de Antofagasta* that has developed a powerful human rights program. This human rights program combined concepts of memory with interactive activities to engage and teach students. From the beginning of the 1990s, during the period of transition back to democracy in Chile, social work schools focused on integrating human rights content into curricula. At first, these were overwhelmingly electives (e.g., 1991 Interdisciplinary Course on Human Rights at the Pontificia Universidad Católica de Chile). Over time, as curricula were adjusted, some universities began to require human rights material, either as independent courses or modules within already-existing courses.

### Theme Two: Creating Collaboration in the Field

In parallel to incorporating human rights content into social work curricula, the second theme that emerged was *creating collaboration in the field*. One social worker interviewed about their experience practicing during the dictatorship stated:

… I was lucky because at the time I studied, we formed the Social Worker collective and an alternative organization. I went to all of those things, then, so it was really different from what the kids are going through today. Because for us, at that point in time, everything related to social work was a challenge, and we wanted to learn techniques to educate and mobilize people, which were not taught to us […] So I think that my education, even though those were very difficult years, it was really deep in this sense. Because we would go to the collective, there on Chile - España Street, we would go to the classes at the alternative organization (testimony 7, compiled in 2012).

The social work collective was not the only entity engaged in reflexive work. Among non-profit organizations, several addressed and reflected about social action during this period, sought to improve labor conditions, health, and overall holistic well-being of people while also encouraging organization of groups of solidarity and promotion of human rights. There are numerous documents that evidence new perspectives that were introduced during this period and that led professionals to recommit themselves to those underserved. Furthermore, new perspectives allowed professionals to integrate concepts from other disciplines, such as international collaboration, businesses, and/or productivity.

**Fig. 1:**
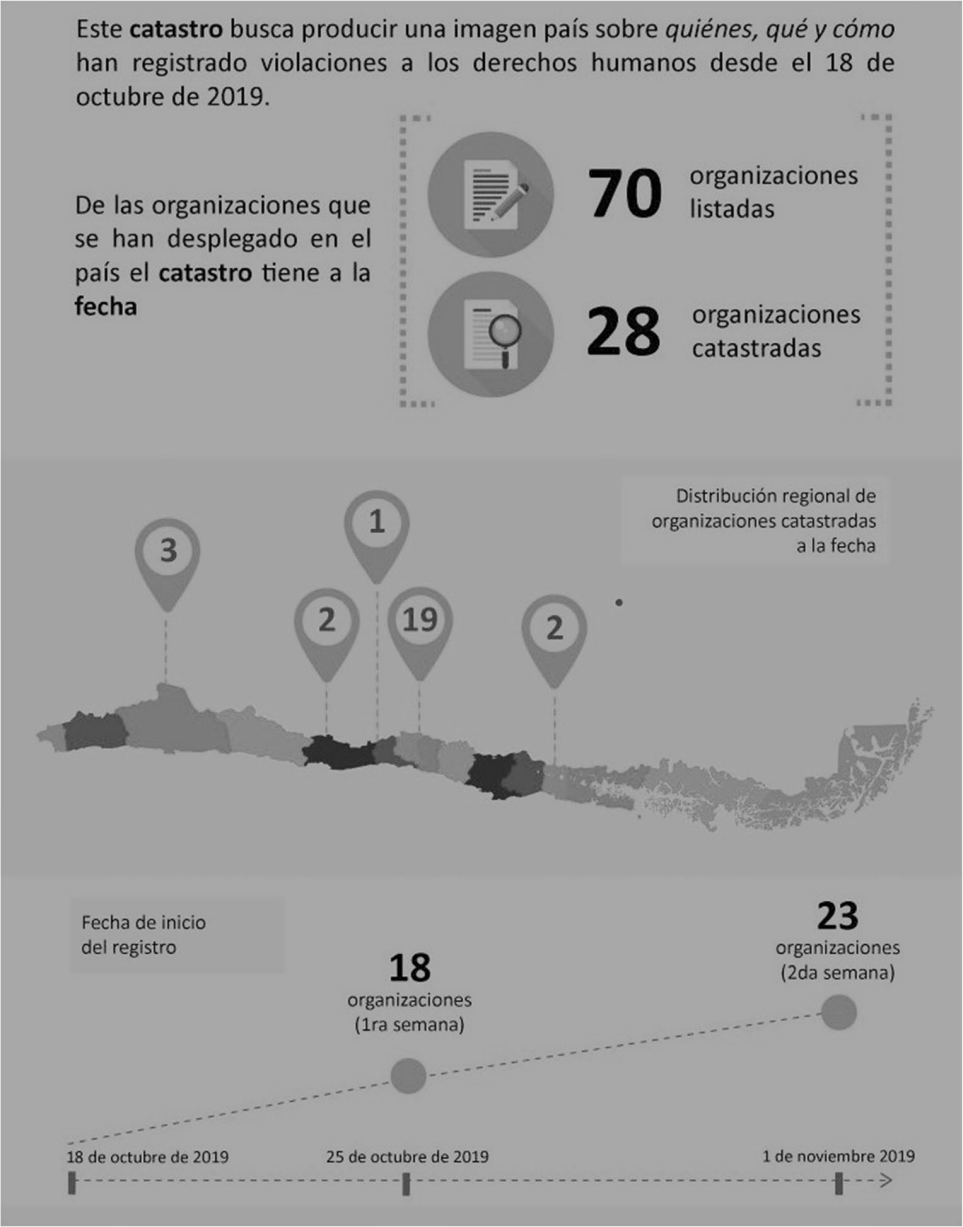
Organizations involved in registering human rights violations in Chile, 2019. Source: Catastro Foro No +, January 2020

With the current unrest and repression that began in October 2019, collaboration in the field has also emerged with several organizations and commissions partnering to document and register human rights violations. Figure 1 below displays the number of organizations or entities in the different regions of Chile that have begun formal processes to compile narratives.

As seen in Fig. 1, a total of 70 organizations have formally compiled narratives and registries of human rights violations since the civil unrest started in 2019. Many of these have involved social workers, and some, such as the commission at the *Universidad de Chile*, have included social work students as a means of providing hands-on practice and support. In the testimonies collected between October and December 2019, participants also mentioned “more solidarity because we are making sure everyone in our communities is okay.” Several of these testimonies mentioned collective resources or organizations that were providing support to those impacted by human rights violations, such as various hospitals or clinics, the Red Cross, neighborhood associations, and student groups at universities, among others. Thus, collaboration in the field consisted both of formal collectives or partnerships, as well as informal support via neighbors and other protestors.

## Discussion

At the beginning of the 1990s, Chile began a transition back to democracy that involved restituting civil, political, and human rights to citizens. This has been an ongoing process, and the recent situations of unrest and police brutality and repression experienced from October 2019 to the present highlight the continued need to address inequalities and continued human rights violations. While some of the strategies that social workers described may not be relevant to the current social and economic context, others have been adapted to advocate for human rights from a holistic perspective. For example, social workers have been instrumental in reinstating commissions to gather testimonies of human rights violations over the past several months. This aligns with strategies social workers utilized during the dictatorship, such as interviews and service provision (individual or groups) with families affected by severe human rights violations. Forty-six years later, social workers have turned to these strategies and mechanisms again to gather information about new human rights violations during the period of social and political crisis. Other strategies that participants described include social work interventions to protect clients that experience domestic, social, or state-sanctioned violence. Given social work’s commitment to social justice and equity, it is in a unique position to develop protocols and policies to serve the needs of individuals who have experienced state-sanctioned violence or police brutality.

### Implications for Social Work Practice

Between 1973 and 1989, social workers denounced and documented violations of human rights in Chile. These reports based on individual interviews became a crucial tool to discover the truth about what happened during the Chilean dictatorship (Morales 2010). Over the course of the past four decades, technological advances have changed the way we gather information, as have the ways we coordinate professionals, student interns, and volunteers to use a human rights approach to social work practice. For example, many organizations in October 2019 created online links to report human rights violations during the unrest (INDH 2019). Nonetheless, with technological advances, it is increasingly important to protect confidentiality and dignity of individuals as they give testimony and narrate their experiences with traumatic experiences. In the case of human rights violations, this is particularly important and social work practitioners and researchers should seek participant consent, establish rapport, and make sure no identifiable information can be gleaned, given potential repercussions or greater victimization. Although social work has been slow to adopt technology (Berzin and Coulton 2018), harnessing technology and utilizing it to address social issues has been recognized as one of twelve grand challenges for social work (Fong et al. 2018). Thus, the advances in technology between the dictatorship and current civil unrest in Chile provide unique opportunities to document human rights violations and raise awareness, something that was not as feasible during Pinochet’s regime.

The use of technology has also emerged in the response and new *toque de queda* in response to COVID-19 in Chile. Again, the government has taken measures to restrict personal liberty, which has led to accusations of human rights violations against those living in poverty, experiencing homelessness, or other vulnerable populations who might not be able to remain home. Thus, as new crises emerge and governments respond, social work practitioners should consider how certain measures may impact human rights or further isolate already-vulnerable populations.

In parallel, as social workers, it is important to raise awareness of these situations and develop strategies to make state-sanctioned violence visible to national and international agencies. In fact, social work is uniquely positioned to address issues of social justice, advocate human rights practice across levels, and support processes of reparation and social justice for victims of human rights violations. Reflections from these experiences are key to replant interventions and research on intergenerational memory. Furthermore, these experiences with human rights intervention and memory are crucial to include in social work formation to prepare future practitioners for issues in the field and in practice settings.

### Implications for Social Work Education

Prior research has supported reflective practice and self-reflection as key components of social work (Sánchez 1990; Saball and Valdés 1990). Reflective practice can also be utilized to critically assess research, theories, and principles that have guided social services since the return to democracy at the end of the twentieth century. In the thirty years since the return to democracy, the narratives and themes that emerged in this study have been given new meaning in light of the recent unrest and protests in Chile. As such, it is increasingly important to integrate human rights into social work pedagogy and prepare students to critically reflect on situations that may arise in field and champion human rights practice. Furthermore, social work curricula could include self-reflection as an anti-oppressive practice, to analyze one’s own positionality and how future practitioners can resist reproduction of dominant power relations (Danso 2015; Heron 2005). By modeling critical reflection and providing pedagogical space to process this in the classroom, social work students can learn to identify patterns of oppression that may lead to human rights violations and work to address these in practice.

Registries, documentation, and reporting situations of abuse have been key in other challenging situations such as the deaths of children and adolescents in state custody (INDH 2018), as well as in identification and support of human trafficking survivors, repression of indigenous communities, or of migrants. In the current context of unrest that started in October 2019, human rights have become a focus again—this time intertwining three generations (Luño 2013). This has also included a human rights focus in social politics and debates about social welfare systems (Rubilar and Grau 2017) and its connection to social, economic, and cultural rights (Morales 2015). As human rights have become more central, social workers have had new challenges in incorporating a human rights approach in their daily practice, policies, and international standards (Álvarez et al. 2006). Moreover, human rights have slowly been integrated into the country since the beginnings of the twenty-first century as more policies reflect an idea of universal rights and equity (Abramovich 2006; Cunill 2010). As Leyton and Muñoz (2016) state “we begin to question how and why people have different opportunities and rights, and how this can make it difficult to hear their voices” (p. 50). Based on this perspective, it is important to teach students how to advocate so that political, social, and cultural rights are guaranteed for all social groups, even—and especially—in contexts where there have been serious human rights violations, such as during the dictatorship or the current unrest. This requires a basic assumption that there are certain unalienable human rights and a wider conceptualization of human rights that are applicable to everyone, including groups previously excluded, such as migrants, people experiencing homelessness, and survivors of exploitation or trafficking (Galaz and Guarderas 2017).

Global social work education has integrated these dimensions of human rights, both formally and informally, in response to serious human rights violations. Yet, despite our profession’s commitment and foundation on social justice and equity, students and academics alike were blatantly under-prepared to confront the violence that erupted in October 2019. As such, they sought guidance from historical memory and support from the field, and from groups such as professional associations and colleagues that advocated for human rights during the dictatorship. Thus, recent events have evidenced a need for a more in-depth formation in human rights so that social work practitioners are prepared to guarantee human rights and integrate this perspective into daily practice, rather than responding to crises as they emerge. In fact, a recent study conducted by Reyes et al. (in press) concluded that there were ongoing challenges to integrate human rights material into higher education in Chile. Indeed, they examined 24 schools of social work at universities across the country, concluding that 58.3% had one specific human rights course. The recent events in Chile highlight the importance of integrating more human rights content into social work curricula across the world—not just in countries with recent histories of human rights violations. This echoes recent calls to explicitly mention human rights as a driver of our profession (Mapp et al. 2019).

### Limitations

While this study provides insight into human rights in social work during two critical periods in Chile, there are some limitations. First, this sample focuses on Chilean social workers who were part of two generations particularly impacted by human rights violations. As such, it is not necessarily generalizable to other countries or contexts. Furthermore, two of the three authors had personal experience with human rights violations during the dictatorship; it is possible that the authors own experiences might have impacted the findings. Nonetheless, the authors incorporated strategies to reduce bias, including bracketing, reflecting on one’s own experience, utilizing audit trails, and consulting with the third author who did not have this personal experience. Finally, the authors did not collect demographic information of the participants in the longitudinal study. Although this was a decision made to protect participant confidentiality, particularly as some discussed historic situations of state-sanctioned and state-perpetrated violence, it does limit the transferability of findings as we do not have the proportion of males to females that participated, age range, highest level of educational attainment, etc.

### Conclusion

This article presented different ways that social workers have integrated human rights into education and practice during two historic periods separated by thirty years in Chile: the military dictatorship (1973–1989) and the social movements during the second half of 2019. These practices are particularly important for social work students and practitioners in other countries that may have less recent experience addressing human rights violations within their borders. Therefore, this article presents a framework for gathering testimony, documenting human rights violations, and giving voice via narratives of those who have experienced and survived state-sanctioned and state-perpetuated violence. The notion of universal, unalienable human rights aligns with social work’s mission to pursue social justice and equity and is particularly relevant given the current social and political context in Chile and in light of anti-immigrant, xenophobic, and nationalistic rhetoric around the globe.

As our profession begins to confront these global inequities, there are lessons to be learned from cases like Chile where social workers have been on the frontline of addressing human rights violations. Social work has always focused on dignity and social justice, but as the world becomes increasingly globalized, our profession may have more instances where we confront human rights violations and issues abroad. Beyond social work practice, this also implies integrating human rights into social work research. These are the main directions for social work that the authors plant from the lessons learned from the discipline in Chile, and we invite other professionals to discuss these challenges and demands for global social work.

## Data Availability

Housed at the Institutional Review Board (IRB) FACSO Universidad de Chile http://www.facso.uchile.cl/facultad/presentacion/107053/comite-de-etica-de-la-investigacion
